# Effects of Electroacupuncture at BL60 on Formalin-Induced Pain in Rats

**DOI:** 10.1155/2012/324039

**Published:** 2012-04-05

**Authors:** Kyung-Ha Chang, Ran Won, Insop Shim, Hyejung Lee, Bae Hwan Lee

**Affiliations:** ^1^Department of Physiology and Brain Korea 21 Project for Medical Science, Yonsei University College of Medicine, Seoul 120-752, Republic of Korea; ^2^Division of Health Science, Department of Biomedical Laboratory Science, Dongseo University, Busan 617-716, Republic of Korea; ^3^Acupuncture and Meridian Science Research Center, Kyung Hee University, Seoul 130-701, Republic of Korea

## Abstract

Acupuncture was used to treat symptoms of pain in the ancient orient. The present study was conducted to determine the effects of electroacupuncture (EA) at the BL60 acupoint on male Sprague-Dawley rats. Each rat received EA at BL60 acupoint before formalin injection. Behavioral responses were recorded using a video camera and c-Fos immunohistochemistry was performed thereafter. Treatment of EA at BL60 significantly inhibited flinching behavior and c-fos expression induced by formalin injection into the paw, compared to a control group. These results suggest that electroacupuncture at BL60 acupoint may be effective in relieving inflammatory pain.

## 1. Introduction

Acupuncture was used to treat symptoms of pain in the ancient orient. Currently, acupuncture has garnered increasing interest as a therapeutic method for treating pain. Recently, electroacupuncture, applying electrical stimulation, is being actively studied. Electroacupuncture was developed to resolve the problems of manual acupuncture, that is, the inconvenient twirling procedure and the difficulty of maintaining constant frequency [1].

The BL60 (Kunlun) acupoint has been shown to be involved in visual information processing [2]. Deadman et al. [3] reported that acupuncture stimulation at BL60 acupoint was effective in treating eye disorders as well as head disorders. Studies indicated that the BL60 acupoint has analgesic effects on chronic low back pain [4] as well as hind limb pain [5]. Li et al. [6] reported that bilateral EA treatment at both BL60 and ST36 acupoints was effective in alleviating inflammatory pain. However, there have been no reports that electroacupuncture at BL60 by itself has an analgesic effect on inflammatory pain.

Inflammatory pain is known to cause abnormality in the nervous system, thereby causing consistent pain [7]. Formalin has been widely used in experiments with animal models of inflammatory pain because of several strong points: (1) it induces behaviors that can be easily observable [7]; (2) the responses to the moderate and continuous pain can be measured [8, 9]; (3) anesthesia is not necessary, so that the behaviors of freely moving animals can be observed [8, 9]. The early phase of pain responses after formalin injection is due to the direct injury of tissues, reflecting nociceptive pain, while the late phase is due to peripheral inflammation and central sensitization [10].

One of the methods used to measure pain and analgesia in animal experiments is the immunohistochemical detection of the c-Fos protein encoded by c-fos, an oncogene. The c-fos gene is an immediate early gene and is rapidly expressed in neurons of the central nervous system when nociceptive stimuli are applied to a peripheral area; for this reason c-Fos is currently widely used as a marker of pain [11, 12].

The present study was performed to determine if electroacupuncture at the BL60 acupoint would alleviate nociceptive pain in the early phase and inflammatory pain in the late phase of responses to formalin injection. In order to determine the effects of electroacupuncture on formalin-induced pain, a behavioral test and c-Fos immunohistochemical study were conducted.

## 2. Methods

### 2.1. Animals

 Male Sprague-Dawley rats weighing about 250~300 g were used in this study. The animals were housed in cages equipped with the barrier system for SPF (specific pathogen free) animals. The system automatically maintained proper temperature (22 ± 2°C), humidity (50 ± 10%) and lighting (12 hr of light/dark). The beddings of the cages were regularly changed (twice a week). All animal experiments were approved by the Institutional Animal Care and Use Committee of Yonsei University Health System.

### 2.2. Electroacupuncture

 A stainless steel needle (diameter 0.25 mm; length 15 mm; Dongbang Acupuncture Inc., Boryeong, Republic of Korea) was used for electroacupuncture [13]. The needle was connected to a wire for better direct application of electric stimuli. The accurate application of electrical stimulation was confirmed in a preliminary study with the experimental animals. The acupoint selected for acupuncture was the BL60 acupoint, which is located at the ankle joint level between the external malleolus and tendo calcaneus in the hind limb [5].

The experiment was designed to determine the efficacy of pretreatment of electroacupuncture (acupuncture treatment before formalin injection) applied to the BL60 acupoint ipsilateral to formalin injection. For this purpose, the animals were divided into three groups: Group 1 was injected with formalin only (Formalin), Group 2 was treated with electrical stimulation by electroacupuncture before formalin injection (EA-For), and Group 3 was treated with needle insertion at the acupoint before formalin injection, but was not treated with real electrical stimulation (Sham-For).

All the animals received inhalation anesthesia of 2% enflurane (95% O_2_/5% CO_2_) prior to electroacupuncture and/or formalin injection. For the EA-For group, which was to receive electroacupuncture at the BL60 acupoint, electrical stimulation was applied using an electrical stimulator (A385, World Precision Instruments, Sarasota, FL, USA) and the Pulsemaster (A300, World Precision Instruments, Sarasota, FL, USA) at 1 Hz (2 ms pulses, 3 mA) for 10 min [14, 15]. The depth of insertion was 2-3 mm. The Sham-For group was kept for 10 minutes with the needle inserted at the BL60 acupoint but no electrical stimulation was applied. The Formalin group was kept under the inhalation anesthesia without any treatment before formalin injection. After electroacupuncture, the animals were kept awake for 10 min in order to acclimate to the test environment.

### 2.3. Behavioral Test

 Formalin is an aqueous solution of formaldehyde (37%). A dilution of formalin in saline was used for the experiments herein: 50 *μ*L of 5% formalin was used for injection, which is widely used to induce maximum pain response [16, 17], while avoiding adverse phenomena, such as ceiling effect, backward walking, and freezing [16, 17].

After electroacupuncture, the experimental animals received formalin injection beneath the left plantar skin using a 29 gauge insulin syringe. Then, the animals were immediately put in an observatory chamber (46 × 26 × 20 cm) and video-recorded for 60 min. The pain behaviors of the rats were analyzed by counting the flinching frequency of formalin-injected paws (number of flinches as 5 min passes) throughout the recording time. After video-recording, the rats were immediately subjected to c-Fos immunostaining.

### 2.4. c-Fos Immunohistochemistry

Normal rats as well as the three groups of rats above were used for c-Fos immunohistochemical study in order to observe changes in c-Fos immunoreactivity by comparing the experimental groups with normal rats. Under anesthesia with 25% urethane (1.25 g/kg, i.p.), the experimental animals were perfused through with 0.9% saline and 4% paraformaldehyde (in 0.1 M phosphate buffer, pH 7.2), and the L5 spinal cord section was removed. The removed tissues were fixed in 4% paraformaldehyde (in 0.1 M phosphate buffer, pH 7.2) for 4 hr at 4°C and then kept in 30% sucrose solution overnight at 4°C. The tissues were cut into 50 *μ*m-thick slices using a Cryocut Microtome (Microm/HM500V, Walldorf, Germany). The total length of the L5 area was about 4000 *μ*m, so that 80 slices of 50 *μ*m thickness were obtained, every 5th slice of which was selected for study for a total of 16 slices. These 16 slices were subjected to free floating staining in the 24-well plates containing 1x phosphate-buffered saline. The tissues reacted in a solution of 30% methanol with 1% H_2_O_2_ for 30 minutes, followed by reactions in a solution with 3% normal goat serum (NGS), 1% bovine serum albumin (BSA), and 0.3% Triton-X for 30 min; and then, the c-Fos antibody (c-Fos anti-rat polyclonal IgG, 1*：*500, Santa Cruz Biotechnology, Santa Cruz, CA, USA) was treated as the primary antibody and the mixture was kept overnight at 4°C. Then, biotinylated anti-rabbit IgG (1 : 200, Vector, Burlingame, CA, USA) was treated as the secondary antibody and the mixture was allowed to react at room temperature for 2 hr, followed by treatment using the ABC kit (Vector, Burlingame, CA, USA) for 1 hr. 3,3-diaminobenzidine tetrahydrochloride (DAB, Sigma, St. Louis, MO, USA) was used for staining and the degree of staining was checked using a microscope (BX40 microscope, Olympus, Tokyo, Japan).

The number of the c-Fos positive neurons was obtained by calculating the mean number of neurons in 4 slices out of the 16 slices that went through the immunohistochemical procedure. A microscope (BX40, Olympus, Tokyo, Japan) was used at a magnification of 10x to check the lamina on the ipsilateral site to the formalin injection, in order to distinguish between lamina I-II and III–VI areas [18]. The MetaMorph software (ver. 4.6, Universal Imaging, Downingtown, PA, USA) was used to count the stained neurons, along with a microscope (BX51, Olympus) mounted with a CCD camera (Cool SNAP Photometrics, Roper Scientific, Tucson, AZ, USA) at a magnification of 10x.

### 2.5. Statistical Analysis

The SPSS 15.0 program (SPSS Inc., Chicago, IL, USA) was used to compare the pain behaviors and the number of the c-Fos positive neurons in each experiment. Data were presented as mean ± SEM, and statistical significance was given when *P* values were less than 0.05. As for the observation of behavioral responses, the flinching frequencies of each group, divided into the early phase and late phase responses, were analyzed by one-way ANOVA followed by Dunnett's multiple comparison test (2-sided) for post hoc analysis. As for the immunostaining, the spinal cords were classified into lamina I-II and III–VI, and the numbers of c-fos positive neurons were compared by the same statistical method mentioned above.

## 3. Results

### 3.1. Behavioral Test

After formalin injection into the plantar paw, rats typically showed vivid flinching behaviors. These responses were characterized into the early phase (short rise and decay) and late phase (long-lasting for about 1 hr) responses. Thus, we classified the flinching behaviors into two phases and compared the effects of electroacupuncture on each phase of the formalin-induced pain behaviors.

**Figure 1:**
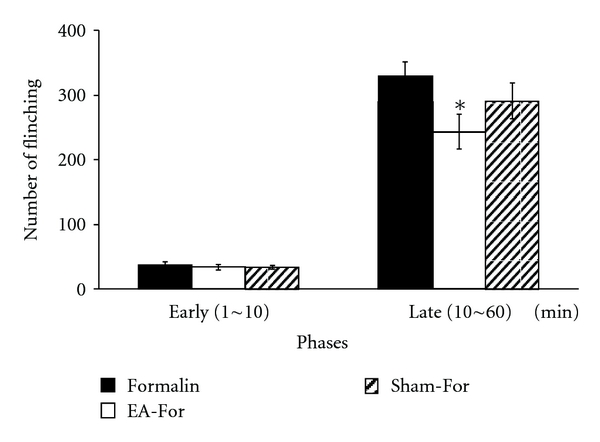
Effects of electroacupuncture on flinching numbers. Bar graphs were divided into early (0–10 min) and late phases (10–60 min). Experimental groups: Formalin formalin injection only group; EA-For electroacupuncture treatment at BL60 before formalin injection; Sham-For acupuncture needle insertion at BL60 but no electric stimulation before formalin injection. In the early phase, there were no significant differences between the groups. However, flinching numbers of the EA-For group were significantly decreased in the late phase. Each bar represents the group mean ± SEM (*P < 0.05).

The frequencies of the flinching behavior in the early phase were as follows: Group 1 37.04 ± 16.76 in the group injected only with formalin (Formalin; *n* = 12); Group 2 33.85 ± 16.24 in the group treated with electroacupuncture before formalin injection (EA-For; *n* = 17); and Group 3 33.96 ± 10.28 in the group with needle insertion before formalin injection, but not treated with electrical stimulation (Sham-For; *n* = 13). As shown in Figure 1, flinching behaviors did not show any significant difference in the early phase (*F*_2,31_ = 3.197, *P* > 0.05).

The frequencies of the flinching behavior in the late phase were as follows: Group 1 329.88 ± 73.56 in the group injected only with formalin (Formalin; *n* = 12); Group 2 243.44 ± 109.33 in the group treated with electroacupuncture before formalin injection (EA-For; *n* = 17); and Group 3 291.42 ± 99.50 in the group with needle insertion before formalin injection, but not treated with electrical stimulation (Sham-For; *n* = 13). The EA-For group showed statistically significant decrease in pain response behaviors compared to the Formalin group (*F*_2,31_ = 5.017, *P* < 0.05; one-way ANOVA followed by Dunnett's multiple comparison) (Figure 1). These data showed that the pre-treatment with electroacupuncture at BL60 significantly inhibited flinching behavior, compared to the control group.

### 3.2. c-Fos Immunohistochemistry

**Figure 2:**
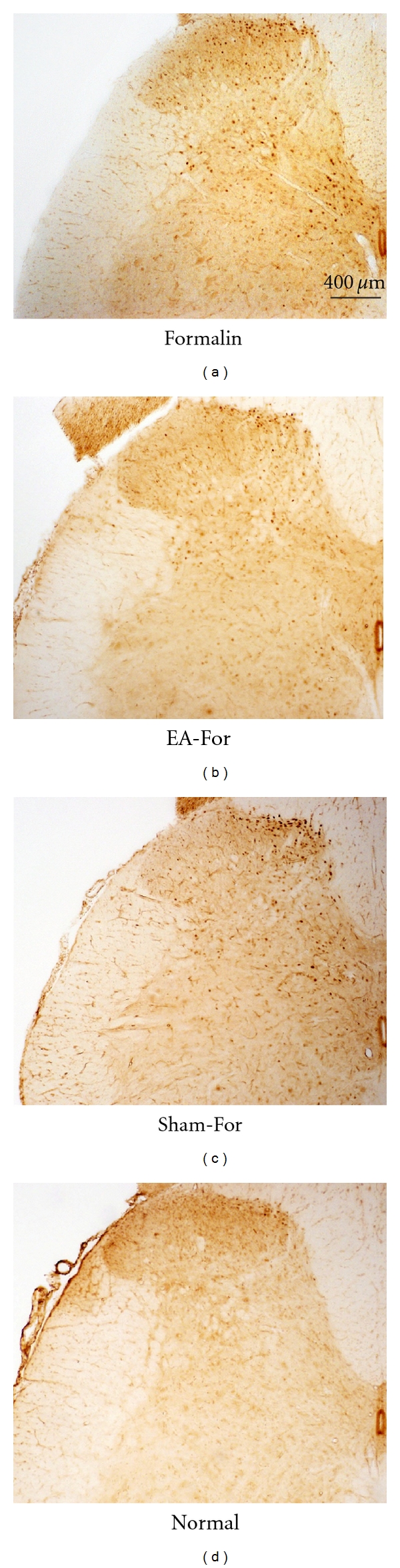
Representative photographs of c-Fos positive neurons in the spinal cord dorsal horn after electroacupuncture. (a) Formalin: formalin injection only group. (b) EA-For, electroacupuncture treatment at BL60 before formalin injection. (c) Sham-For, acupuncture needle insertion at BL60 but no electrical stimulation before formalin injection. (d) Normal: no treatment group.

 The number of c-Fos positive neurons in the dorsal horn was counted separately for superficial layers (lamina I-II) and deep layers (III–VI). The representative photographs of the c-Fos positive neurons of the individual groups are shown in Figure 2. As shown in Figure 2, the EA-For group showed a remarkable decrease in the number of c-Fos positive neurons, compared to the Formalin group.

**Figure 3:**
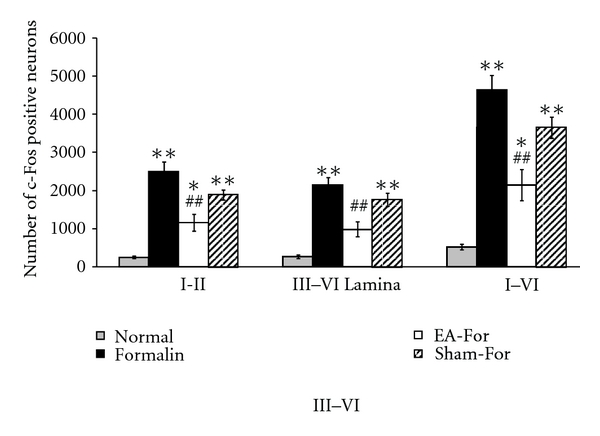
The number of c-Fos positive neurons in different groups. C-Fos positive cells were counted by MetaMorph software in lamina I-II and lamina III–VI at the L5 segment of the spinal cord ipsilateral to the site of formalin injection. Experimental groups were classified as follows: Normal: no treatment group; Formalin: formalin injection only group; EA-For, electroacupuncture treatment at BL60 before formalin injection; Sham-For, acupuncture needle insertion at BL60 but no electric stimulation before formalin injection. In each region, there was a significant decrease of c-Fos positive neurons when electroacupuncture was treated at BL60 compared to the Formalin group. On the other hand, in a comparison between Normal group and the other groups, there were notable increases in c-Fos positive neurons in the EA-For group and Sham-For group. Each bar represents the group mean ± SEM (*P < 0.05, **P < 0.01 for comparison between Normal group and all the other groups, one-way ANOVA followed by Dunnett's post hoc multiple comparison: ^##^ *P* < 0.01 for comparison between Formalin group and EA-For or Sham-For group, one-way ANOVA followed by Dunnett's post hoc multiple comparison).

In order to compare the level of c-Fos immunoreactivity in the Formalin group Group 1 with pain-free animals, a normal naïve animal group Group 0 was added to this study. The numbers of c-Fos positive neurons in lamina I-II were as follows: Group 0,  250 ± 30.68  in the group with no treatment (Normal; *n* = 4); Group 1,  2501 ± 255.54  in the group injected only with formalin (Formalin; *n* = 7); Group 2, 1163.12 ± 219.16 in the group treated with electroacupuncture before formalin injection (EA-For; *n* = 8); and Group 3,  1893 ± 132.91  in the group with needle insertion before formalin injection, but not treated with electrical stimulation (Sham-For; *n* = 10) (left in Figure 3).

For lamina III–VI, the numbers were 268.75 ± 45.38 (Normal; Group 0), 2143.57 ± 200.389 (Formalin; Group 1), 985.62 ± 195.79 (EA-For; Group 2), and  1762 ± 171.84  (Sham-For; Group 3), respectively (middle in Figure 3). The total numbers of the c-Fos positive neurons in lamina I through VI were 518.75 ± 71.97, 4645 ± 372.30, 2148.75 ± 406.42, and 3655 ± 274.78, respectively (right in Figure 3). When the Normal group Group 0 was compared to each group, the Formalin group Group 1 and the Sham-For group Group 3 showed a statistically significant increase in the number of the c-Fos-expressed neurons in each lamina (I-II: *F*_3,17_ = 23.316; III–VI: *F*_3,17_ = 23.546; I–VI: *F*_3,17_ = 33.101, *P* < 0.05; one-way ANOVA followed by Dunnett's multiple comparison). When the Formalin group Group 1, the EA-For group Group 2, and the Sham-For group Group 3 were compared, however, the EA-For group Group 2 showed a statistically significant decrease in the number of c-Fos positive neurons compared to the Formalin group Group 1 in each lamina (I-II: *F*_2,14_ = 14.711; III–VI: *F*_2,14_ = 15.647; I–VI: *F*_2,14_ = 21.340, *P* < 0.05; one-way ANOVA followed by Dunnett's multiple comparison). These results showed that the pretreatment of EA at BL60 significantly inhibited c-Fos expression induced by formalin injection into the paw, compared to control group.

## 4. Discussion

Acupuncture has been used in Western medicine as well as Oriental medicine as an alternative treatment for pain-related disorders. In particular, electroacupuncture was developed to overcome the disadvantages of manual acupuncture, that is, the inconvenient twirling procedure and the difficulty of maintaining constant frequency.

Of inflammatory pain models, a formalin model was used in the present study in order to compare distinct biphasic nociceptive behavioral responses at both early and late phases after formalin injection. Electroacupuncture was pretreated before formalin injection in order to compare the effects of EA stimulation on both phases of formalin-induced pain but was not posttreated because the effects of electroacupuncture stimulation on both the early and late phases only would be unobservable if electroacupuncture was applied after formalin injection, even though post-treatment resembles a natural clinical condition.

In the present study, EA stimulation with low-frequency (1 Hz, 3 mA) at BL60 acupoint inhibited flinching behaviors in the late phase, but not in the early phase after formalin injection in rats. In electroacupuncture treatment, it has been shown that low-frequency stimulation produces prolonged analgesic effects relatively late in electroacupuncture; however, high-frequency stimulation produces short-lasting analgesic effects immediately after initiation of electroacupuncture [1]. Similarly, Lao et al. [18] showed that high frequency electroacupuncture produces the most potent anti-hyperalgesia in the early stage of complete Freund's adjuvant- (CFA-)induced hyperalgesia, while low-frequency electroacupuncture produces a prolonged inhibitory effect to reduce hyperalgesia.

As used in the present study, electrical stimulation with the intensity of 3 mA is known to be the maximum that conscious animals can withstand [18]. It was observed that the muscles around at the tip of the experimental animals' feet were trembling when electrical stimulation was applied with the intensity of 3 mA. It was reported that this intensity of electroacupuncture stimulation has a therapeutic effect in inflammatory pain models [18].

According to Chang et al. [19], analgesic effects were observed at both early and late phases when electroacupuncture (2, 10, 100 Hz frequency, 3 mA intensity, for 5 min) was applied bilaterally at the ST36 (Zusanli) acupoint in mice. Kim et al. [17] reported that bilateral electroacupuncture (2 ms, 10 Hz, 3 mA, for 30 min) at both HE7 (Shenmen) and PE7 (Daling) acupoints produces analgesic effects lasting nearly 1 hour in both early and late phases of formalin-induced pain in rats. These results indicate that the pain-relieving effects of electroacupuncture persist after EA stimulation cessation.

However, our behavioral results herein are not fully consistent with other studies [17, 19] that observed analgesic effects of EA in both early and late phases. The discrepancy may be attributable to differences in the acupoints treated, parameters in electroacupuncture stimulation, laterality, or experimental animals.

However, aspirin [20], nonsteroidal anti-inflammatory drugs (NSIADs) such as indomethacin and naproxen [21], compound 48/80, a histamine and serotonin depleter [9], and spinal anesthesia [22] can reduce late phase but not early phase formalin-induced pain. In relation to these studies, our study suggests that electroacupuncture stimulation at BL60 acupoint may be effective in relieving persistent inflammatory pain rather than in relieving acute pain produced by immediate activation of nociceptors in formalin test models.

There have been numerous studies that observed the pain-relieving effects of acupuncture [13, 16, 17, 23–25]. To our knowledge, however, there are no reports except for Kim et al. [26] and Zou et al. [27] to study the pain-relieving effects of acupuncture at the BL60 acupoint. Disappointingly, Kim et al. [26] did not find any analgesic effect with acupuncture at BL60. According to Zou et al. [27], patients with lumbar intervertebral disc herniation treated with EA combined with acupoint-injection at L4 Jiaji (EX-B2), L5 Jiaji (EX-B2), Zhibian (BL54), Huantiao (GB30), Yanglingquan (GB34), Wizhong (BLA0), and Kunlun (BL60) showed significant improvement of pain, compared to a control group treated with electroacupuncture only. However, their study did not test the effects of electroacupuncture at BL60 acupoint itself. Therefore, our study may be the first to show the analgesic effects of electroacupuncture at BL60 acupoint in inflammatory pain.

Since Hunt et al. [28] reported c-Fos expression in response to peripheral nociceptive stimuli, c-Fos has been used as a neuronal marker of pain. Even though the role and significance of c-Fos expression in pain transmission is not fully understood [29], the remarkable correlation between the distribution of c-Fos positive neurons in the superficial layers (laminae I and II) of the lumbar spinal cord after formalin injection and the spinal distribution of pain-related afferent fibers innervating the plantar surface of the hind paw [30–32] suggests that the Fos protein may be expressed as a result of postsynaptic activation by pain-related afferents. In deep layers, few neurons receive direct pain-related afferents while most neurons receive convergent inputs from superficial layers [33], suggesting that Fos expression at the spinal cord reflects activation of second order neurons related to transmission of pain. Therefore, identification of c-Fos expression in the spinal dorsal horn, associated with paw flinching responses induced by formalin injection, can provide morphological anatomic evidence of pain and/or analgesia [34].

In relation to c-Fos expression in formalin-induced pain models, c-Fos seems to be expressed in superficial layers in relation to early phase pain and in deep layers in relation to late phase pain, while overall c-Fos expression appears to be concentrated in superficial lamina I-II [35]. However, in the present study, electroacupuncture treatment at BL60 acupoint reduced the number of c-Fos positive neurons in not only superficial layer laminae I-II but also in deep layer laminae III–VI of the spinal cord at the L4-5 level, compared to a formalin injection only group. This result is consistent with other studies which observed a reduction of c-Fos expression in both superficial and deep layers of the spinal cord dorsal horn in the electroacupuncture-treated groups of a carrageenan-induced pain rat model [16, 36] and a formalin model [17]. Furthermore, it is considered that the mechanism of pain suppression by electroacupuncture at BL-60 acupoint is due to its action at the spinal cord level, but not at the supra-spinal level. Also, the fact that there was a significant decrease in the number of c-Fos positive neurons in all parts of the lamina I-II and III–VI indicated that there is a close relationship between flinching responses and the number of c-Fos positive neurons during the treatment of electroacupuncture.

However, the mechanisms of analgesic effects of electroacupuncture treatment on formalin-induced pain are uncertain. Our results showed significant decrease in flinching response after the pretreatment of electroacupuncture. In our accompanying study, however, there was no significant reduction in licking response after the pretreatment of electric acupuncture (data are not shown). The flinching response is regarded to be a simple flexor reflex which is mediated at the spinal level, while the lifting or licking response is considered to be a more complex reflex which is mediated at the supraspinal level [36, 37]. It has been shown that the number of neurons expressing c-Fos increases when pain-transmitting neurons are activated in the spinal cord [28–33]. Our study showed that the number of c-Fos positive neurons decreased after electroacupuncture stimulation. This suggests that electroacupuncture relieves pain by inhibiting the transmission of pain at the level of the spinal cord.

In the spinal cord, the transmission of pain may be regulated in different ways. For example, activation of descending pain inhibition system from the brain may reduce the transmission of pain [38–40]. According to the gate control theory of Melzack and Wall [41], on the other hand, fast-conducting somatosensory impulses by electroacupuncture stimulation arrive at the spinal cord dorsal horn to inhibit the activity of pain-transmitting neurons of the dorsal horn by blocking the input of pain information conducted from peripheral inputs [42–44]. Otherwise, electroacupuncture stimulation may induce diffuse noxious inhibitory controls (DNICs) in which analgesic effects by electroacupuncture treatment can be diffuse and unspecific, as the pain-relieving effect of electroacupuncture depends on the intensity of stimulation rather than the precise location thereof [45–47]. Nevertheless, the detailed mechanisms of the analgesic effects of electroacupuncture treatment still remain to be determined. In order to elucidate these mechanisms, further studies will be needed.

## 5. Conclusion

The effects of electroacupuncture treatment at the BL60 acupoint (BL60) were examined in experimental animals with inflammatory pain induced by formalin. Judging from the fact that there was suppression of flinching behavior responses and of c-Fos expression upon the treatment of electroacupuncture therein, the treatment of electroacupuncture at BL60 acupoint is very effective in alleviation of inflammatory pain.
